# Genomic Medicine Sweden: Advancing precision medicine at the national level

**DOI:** 10.1111/joim.70129

**Published:** 2026-07-06

**Authors:** Anders Edsjö, Anna Lindstrand, Panagiotis Baliakas, David Gisselsson, Paula Mölling, Mia Wadelius, Åsa Johansson, Hannes Olauson, Hans Ehrencrona, Lovisa Lovmar, Christian G. Giske, Henrik Green, Ann Nordgren, Fulya Taylan, Martin Hallbeck, Erika Tång Hallbäck, Anna Green, Markus Heidenblad, Kina Höglund, Anders Jemt, Carl Mårten Lindqvist, Maria Johansson Soller, Magnus Sabel, Colum Walsh, Hartmut Vogt, Craig E. Wheelock, Martin Bergö, Jens Enoksson, Ann Ekberg Jansson, Cecilia Fagerström, Jan Holgersson, Stephanie Anja Juran, Mats G. Karlsson, Frida Lundmark, Eva Tiensuu Janson, Andreas Muranyi Scheutz, Malin Sund, Päivi Östling, Sofia Gruvberger‐Saal, Marene Landström, Malin Melin, Lars Palmqvist, Richard Palmqvist, Bianca Stenmark, Anna Wedell, Malin Karnå, Katarina Nyström, Tobias Strid, Per Sikora, Maria Johansson, Therese Fagerqvist, Mirja Carlsson Möller, Oskar Frisell, Mats Ulfendahl, Mikaela Friedman, Lucia Cavelier, Valtteri Wirta, Thoas Fioretos, Richard Rosenquist

**Affiliations:** ^1^ Department of Clinical Genetics Pathology and Molecular Diagnostics Skåne University Hospital Lund Sweden; ^2^ Division of Pathology Department of Clinical Sciences Lund University Lund Sweden; ^3^ Department of Molecular Medicine and Surgery Karolinska Institutet Stockholm Sweden; ^4^ Clinical Genetics and Genomics Karolinska University Hospital Stockholm Sweden; ^5^ Genomic Medicine Center Karolinska Karolinska University Hospital Stockholm Sweden; ^6^ Department of Immunology Genetics and Pathology Uppsala University Uppsala Sweden; ^7^ Division of Clinical Genetics Department of Laboratory Medicine Lund University Lund Sweden; ^8^ Department of Laboratory Medicine Faculty of Medicine and Health Örebro University Örebro Sweden; ^9^ Department of Medical Sciences Clinical Pharmacogenomics Uppsala University Uppsala Sweden; ^10^ Department of Clinical Pathology and Cancer Diagnostics Karolinska University Hospital Stockholm Sweden; ^11^ Department of Clinical Genetics and Genomics Sahlgrenska University Hospital Region Västra Götaland Gothenburg Sweden; ^12^ Department of Laboratory Medicine Karolinska Institutet Stockholm Sweden; ^13^ Department of Clinical Microbiology Karolinska University Hospital Stockholm Sweden; ^14^ Department of Biomedical and Clinical Sciences Linköping University Linköping Sweden; ^15^ Department of Forensic Genetics and Forensic Toxicology National Board of Forensic Medicine Linköping Sweden; ^16^ Science for Life Laboratory Linköping University Linköping Sweden; ^17^ Department of Laboratory Medicine Institute of Biomedicine University of Gothenburg Gothenburg Sweden; ^18^ Department of Clinical Pathology Linköping University Hospital, Region Östergötland Linköping Sweden; ^19^ Department of Infectious Diseases Institute of Biomedicine Sahlgrenska Academy University of Gothenburg Gothenburg Sweden; ^20^ Department of Clinical Microbiology Sahlgrenska University Hospital Region Västra Götaland Gothenburg Sweden; ^21^ Clinical Genomics Lund Science for Life Laboratory Lund University Lund Sweden; ^22^ Department of Microbiology Tumor and Cell Biology Clinical Genomics Stockholm Science for Life Laboratory Karolinska Institutet Solna Sweden; ^23^ Clinical Genomics Örebro Science for Life Laboratory Örebro University Örebro Sweden; ^24^ Clinical Research Center Faculty of Medicine and Health Örebro University Örebro Sweden; ^25^ Department of Clinical Genetics Uppsala University Hospital Uppsala Sweden; ^26^ Childhood Cancer Centre Sahlgrenska University Hospital Region Västra Götaland Gothenburg Sweden; ^27^ Department of Pediatrics Institute of Clinical Sciences Sahlgrenska Academy University of Gothenburg Gothenburg Sweden; ^28^ Crown Princess Victoria Children's Hospital Region Östergötland Linköping Sweden; ^29^ Unit of Integrative Metabolomics Institute of Environmental Medicine Karolinska Institutet Stockholm Sweden; ^30^ Department of Respiratory Medicine and Allergy Karolinska University Hospital Stockholm Sweden; ^31^ Department of Medicine, Huddinge Karolinska Institutet Huddinge Sweden; ^32^ Regional Executive Office Region Västra Götaland Gothenburg Sweden; ^33^ Department of Internal Medicine and Clinical Nutrition Institute of Medicine University of Gothenburg Gothenburg Sweden; ^34^ Department of Research Region Kalmar County Kalmar Sweden; ^35^ Department of Health and Caring Sciences Faculty of Health and Life Sciences Linnaeus University Kalmar/Växjö Sweden; ^36^ Department of Clinical Immunology and Transfusion Medicine Sahlgrenska University Hospital Region Västra Götaland Gothenburg Sweden; ^37^ Rare Diseases Sweden Stockholm Sweden; ^38^ Regional Executive Office Region Örebro County Örebro Sweden; ^39^ Lif, the research based pharma companies Stockholm Sweden; ^40^ Department of Medical Sciences Endocrine Oncology Unit Science for Life Laboratory Uppsala University Uppsala Sweden; ^41^ Regional Executive Office Region Stockholm Stockholm Sweden; ^42^ Department of Diagnostics and Intervention/Surgery Umeå University Umeå Sweden; ^43^ Department of Surgery University of Helsinki and Helsinki University Hospital Helsinki Finland; ^44^ Department of Oncology‐Pathology Data Center SciLifeLab Karolinska Institutet Solna Sweden; ^45^ Karolinska Comprehensive Cancer Center Project Unit Karolinska University Hospital Stockholm Sweden; ^46^ Department of Medical Biosciences Umeå University Umeå Sweden; ^47^ Department of Clinical Chemistry Sahlgrenska University Hospital Region Västra Götaland Gothenburg Sweden; ^48^ Centre for Inherited Metabolic Diseases Karolinska University Hospital Stockholm Sweden; ^49^ Region Västra Götaland Gothenburg Sweden; ^50^ Sahlgrenska Academy University of Gothenburg Gothenburg Sweden; ^51^ Department of Precision Medicine Laboratory Region Östergötland Linköping Sweden; ^52^ Core‐Facilities, Bioinformatics and Data Centre University of Gothenburg Gothenburg Sweden; ^53^ Lund University Collaboration Office Lund University Lund Sweden; ^54^ Research and Partnership Support Uppsala University Uppsala Sweden; ^55^ The Swedish Institute for Health Economics Lund Sweden; ^56^ Department of Learning Informatics, Management and Ethics Karolinska Institutet Solna Sweden; ^57^ Regional Executive Office Region Östergötland Linköping Sweden; ^58^ School of Engineering Sciences in Chemistry Biotechnology and Health Clinical Genomics Stockholm Science Life Laboratory KTH Royal Institute of Technology Stockholm Sweden

**Keywords:** cancer, complex diseases, genomic medicine, health economics, microbiology, pharmacogenomics, precision medicine, rare diseases

## Abstract

High‐throughput sequencing has transformed clinical diagnostics of rare diseases (RD), cancer and infectious diseases by enabling the identification of disease‐causing genetic alterations and facilitating individualised treatment and care. In response to these advances, Genomic Medicine Sweden (GMS) was established in 2017 as a national collaborative effort to accelerate implementation of genomics‐based precision medicine within Sweden's regionally organized, publicly funded healthcare system. GMS brings together the seven university healthcare regions and their associated medical faculties, in collaboration with healthcare regions across Sweden, Science for Life Laboratory, patient organizations, industry and governmental agencies. Activities are coordinated through national disease‐specific expert groups, supported by cross‐cutting functions in bioinformatics, health economics, ethics, education and patient engagement. At the operational level, seven Genomic Medicine Centres, embedded at university hospitals, develop and deliver harmonised genomic diagnostics nationwide. The National Genomics Platform provides secure infrastructure for large‐scale data storage, analysis, and national and international data sharing. Following initial project‐based funding, GMS now receives long‐term governmental support. This review describes the national implementation of genomic‐based precision diagnostics, discusses challenges and lessons learnt, and highlights key milestones across disease areas, including whole‐genome sequencing in RD and paediatric cancer, comprehensive genomic profiling of haematological malignancies and solid tumours, pathogen genomics in microbiology, pharmacogenomic testing and emerging applications of polygenic risk scores in complex diseases. Collectively, these efforts have contributed to more than 500,000 genomic tests being performed within Swedish healthcare between 2017 and 2025. Finally, we outline future diagnostic needs and priority areas to ensure sustainable, scalable and equitable access to precision medicine.

## Graphical abstract

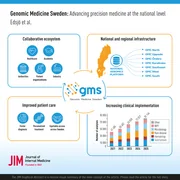

## Introduction

The widespread adoption of high‐throughput sequencing (HTS) in the past decade has transformed biomedical research and clinical practice [1, 2]. These technologies have enabled large‐scale identification of molecular alterations linked to disease, leading to the discovery of numerous genes responsible for rare diseases (RD), a deeper understanding of the genomic complexity of major cancer types and pathogen recognition at unprecedented scale [3, 4, 5]. At the same time, therapies targeting disease‐driving mutations or signalling pathways have advanced molecularly tailored treatments.

As a result, genomic technologies, such as whole‐genome sequencing (WGS) and targeted gene panels, have rapidly moved from research settings into clinical diagnostics and are now widely used in routine diagnostics of RD, cancer and infectious diseases [6, 7, 8]. These approaches enable comprehensive molecular profiling, supporting more accurate diagnosis, risk stratification and individualized treatment. Together, these advances have laid the foundation for precision medicine, in which treatment and care are tailored to each patient's genetic profile [9].

Early national initiatives accelerated the transition of genomics from research into clinical care, exemplified by programs such as Genomics England. Large‐scale sequencing efforts in countries, including England, Denmark and France, demonstrated the feasibility and clinical utility of integrating genomic technologies into publicly funded healthcare systems [10, 11]. Broadly, two implementation models have emerged [12]. The first is a top‐down approach, typically driven by a national strategy with centralized coordination and funding. The second is a bottom‐up model, common in countries with regionally organized healthcare systems [13, 14, 15]. In these settings, regionally based genomic centres have been established and aligned through national coordination to enable collaboration while preserving regional governance.

In this review, we describe Sweden's efforts to establish a national infrastructure for genomic‐based precision medicine within a regionalised healthcare system. We highlight key disease areas where genomic approaches are being implemented and discuss remaining challenges to ensure equitable access. Finally, we outline future directions as genomic and precision medicine continue to expand their role in healthcare.

## Establishment of Genomic Medicine Sweden

Genomic Medicine Sweden (GMS) was initiated in 2017 as a national effort to accelerate integration of genomic technologies into Swedish healthcare and build a sustainable framework for precision medicine [13, 16]. From 2017 to 2025, the initiative was primarily financed through grants from the Swedish innovation agency Vinnova, along with co‐funding from partner organizations, and now receives core funding from the Swedish government. GMS is founded on a partnership between the seven university healthcare regions and the seven universities with medical faculties and collaborates closely with Science for Life Laboratory (SciLifeLab), Biobank Sweden, patient organizations and industry. GMS originated within SciLifeLab's Clinical Genomics platform, which continues to serve as the technical backbone for method and assay development within GMS [13].

The organization is structured around seven national disease‑specific expert groups, that is, RD, solid tumours, haematological malignancies, childhood cancer, microbiology, complex diseases and pharmacogenomics, supported by cross‑cutting working groups in bioinformatics, health economics, industry collaboration, education, ethics and patient engagement. The National Genomics Platform (NGP) enables secure, standardized nationwide sharing of genomics data and provides the core infrastructure required for implementing precision medicine at scale.

**Fig. 1:**
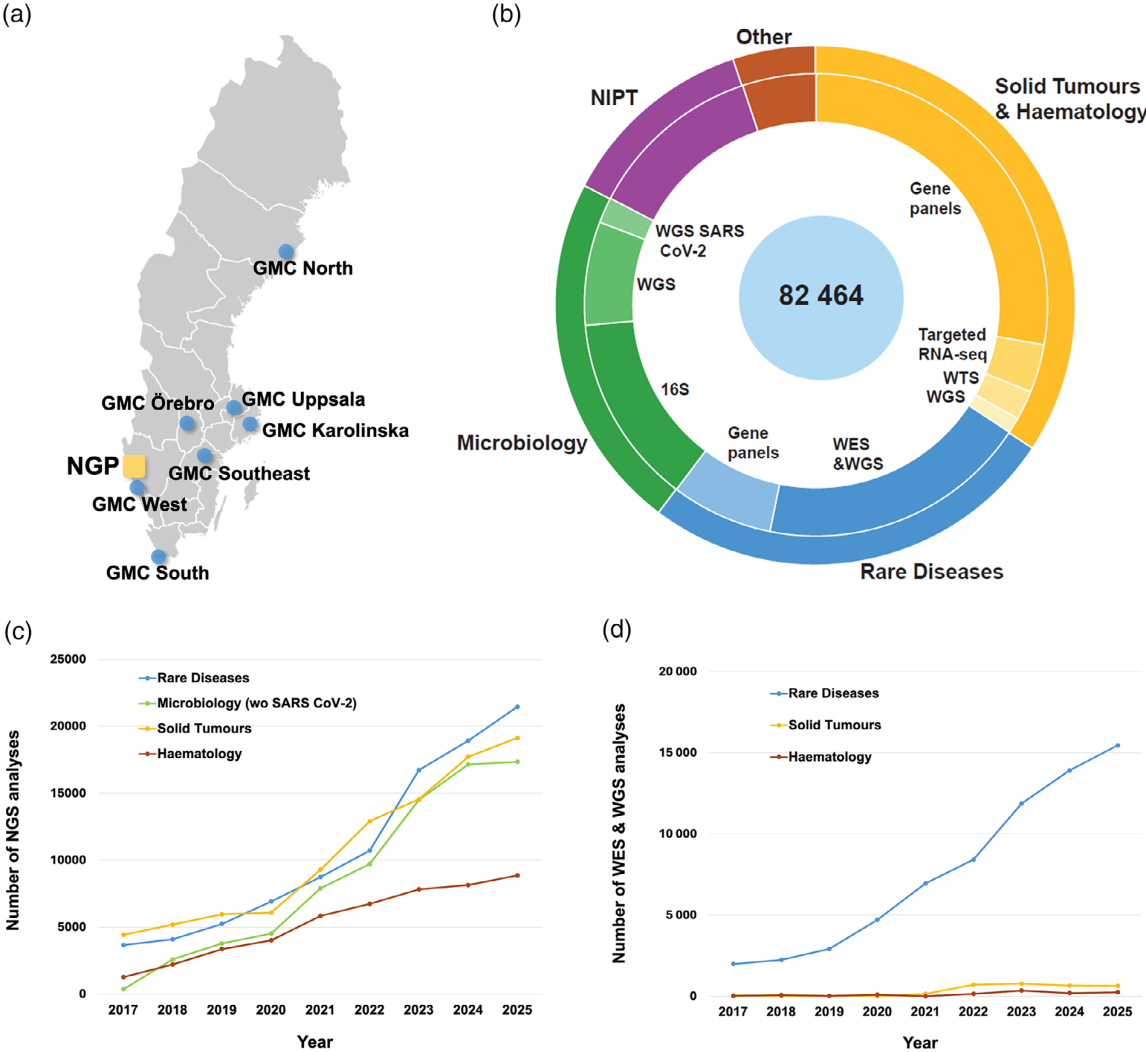
NGS‐based analyses performed within Swedish healthcare. (a) Map showing Genomic Medicine Centres (GMCs) in Sweden and the National Genomic Platform (NGP). (b) Distribution of the total number of NGS‐based analyses (n = 82,464) across different disease areas conducted within Swedish healthcare during 2025. (c) Total number of NGS‐based analyses performed between 2017 and 2025 in rare diseases, haematological malignancies, solid tumours and microbiology. (d) Total number of WGS and WES analyses carried out between 2017 and 2025 in rare diseases and cancer. Current reporting is performed at the national level, as sequencing analyses may be carried out in a different healthcare region from where the patient resides. Integration of patient residence data in future analyses will enable more accurate assessment of regional implementation and utilization patterns. NIPT, non‐invasive prenatal testing; WES, whole‐exome sequencing; WGS, whole‐genome sequencing; WTS, whole‐transcriptome sequencing.

Sweden's healthcare system is organized into 21 healthcare regions responsible for healthcare delivery. To support national implementation of genomic‐based precision medicine, seven Genomic Medicine Centres (GMCs) have been established at the seven university hospitals in Sweden (Fig. 1), in close collaboration with their partner universities. This GMC network ensures harmonized implementation across the country while allowing adaptations to regional needs and strengths. Each GMC also supports the neighbouring non‑university healthcare regions, promoting equitable access to genomic medicine across all regions nationwide.

GMS is governed by a national steering group, with coordination handled by a management group with representation from participating partners. Collaboration extends beyond healthcare and academia to include national infrastructures and stakeholders, including SciLifeLab, Biobank Sweden, patient organizations, Regional Cancer Centres, industry and government agencies, forming a broad innovation ecosystem around precision medicine. Together, the organizational structure, national–regional partnerships and robust laboratory and informatics provide the foundation for sustainable integration of genomic‐based precision medicine into Swedish healthcare.

## Implementation of precision medicine

In the following sections, we illustrate how genomics‐based precision medicine has been developed and implemented across our key diagnostic areas, highlighting representative examples of successful applications. GMS has monitored the number of genomic‐based tests performed in healthcare as an indicator of clinical uptake (Fig. 1). Between 2017 and 2025, more than 500,000 genomic tests were conducted in Swedish healthcare, corresponding to an annual increase of approximately 10%–15% in the last 5 years. It should be noted that decisions regarding the implementation, test directories and reimbursement of genomic diagnostics are made independently within each healthcare region.

### Rare diseases

**Fig. 2:**
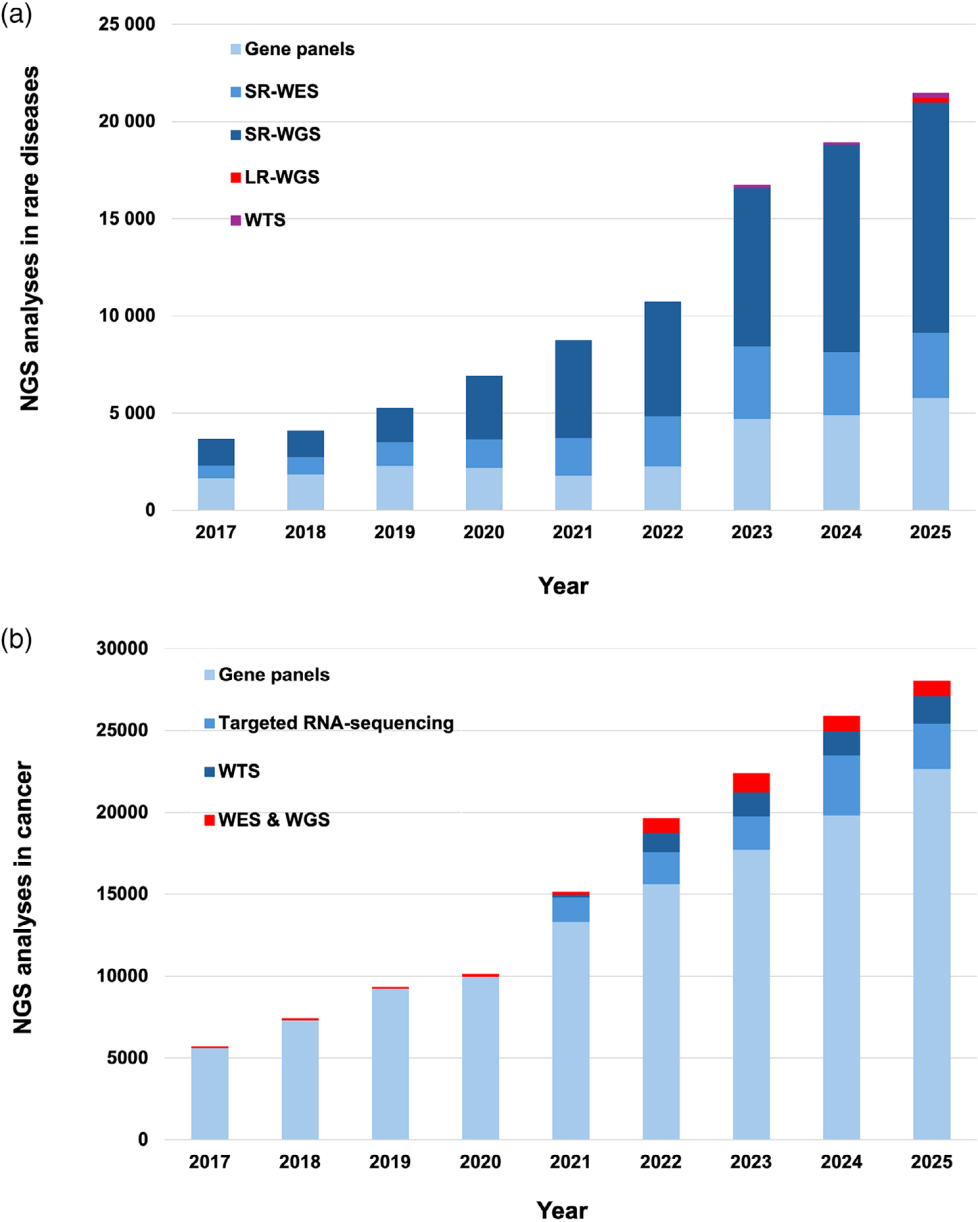
NGS‐based analyses performed between 2017 and 2024 in (a) rare diseases and (b) cancer. LR‐WGS, long‐read whole‐genome sequencing; SR‐WGS, short‐read whole‐genome sequencing; WES, whole‐exome sequencing; WTS, whole‐transcriptome sequencing.

RD affects approximately 30 million individuals in the EU and represents a major public health challenge despite the low prevalence of each individual diagnosis. Patients often undergo prolonged diagnostic odysseys, with sequential testing across specialties and variable access to advanced genomic investigations [8]. Within GMS, the RD Working Group (GMS‐RD) supports the implementation of genomics in routine care, with the long‐term mission of improving diagnostics, including consistent interpretation over time and across healthcare regions. Short‐read WGS has been prioritized with the overall aim of enabling streamlined computational workflows for comprehensive variant detection tailored to an individual's specific symptoms [5, 8]. Implementation has progressed stepwise across Swedish healthcare, and by 2025 >12,000 WGS analyses were performed annually, with sequencing validated at five GMCs (Figs. 1 and 2). Importantly, regions without local sequencing capacity can refer samples for testing and interpretation within the GMS network, supporting more equitable access for individuals with RD across Sweden.

A significant step was the first GMS‐RD strategic pilot project launched in 2020, which served as a nationwide alignment effort, bringing sites together around shared workflows and interpretation practices and accelerating both local implementation and coordinated national development. The pilot focused on individuals with intellectual disability, with or without congenital malformations. In total, 25 patients were included, and trio WGS was performed; clinically relevant findings were identified in 52% of cases, providing diagnoses for 13 individuals and their families.

Key activities to ensure high‐quality clinical WGS nationwide after the pilot have included community‐driven development of a national computational pipeline [17] capable of robust detection of all clinically relevant variant classes, including single nucleotide variants (SNVs)/insertions–deletions (indels), structural variants (SVs) and short tandem repeats (STRs). The overall diagnostic yield is ∼25% for singletons and ∼30% for trios, depending on inclusion criteria [5, 18, 19]. By pooling expertise, GMS‐RD has reduced regional silos, enabled shared learning, and accelerated method development, pipeline updates, cross‐site validation, and consistent interpretation.

A key strategic activity for GMS‐RD has been the development of a clinical genetic database for genotype–phenotype matching, aimed at strengthening interpretation, facilitating case matching and supporting reanalysis as knowledge evolves. Progress has, however, been hampered by legislative and governance differences across regions that complicate data sharing in clinical routines. To address this, GMS‐RD formed a multi‐centre research study that enables structured data sharing between partners and supports collaboration with international initiatives, including the European Beyond 1 Million Genomes effort. In parallel, the study provides a framework for (i) integrated analysis of patients’ genomic and clinical data to identify new disease genes and mechanisms and (ii) provides continuous method development, enabling evaluation of new analytic approaches and the next generation of tools for RD diagnostics. This work has also driven national harmonization, with a common informed consent used across all GMCs for RD research.

To facilitate broad inclusion, GMS‐RD has developed an electronic informed consent (eConsent) solution allowing individuals to log in using electronic ID and consent to research and data sharing [20]. The system also supports registration of legacy paper consents into a unified, searchable database, creating long‐term value for traceability and future research within GMS. Currently, >5000 individuals have been registered, either via direct eConsent or through digitization of collected paper consents.

In 2022, GMS‐RD initiated a national long‐read WGS pilot focused on digital karyotyping and breakpoint‐level resolution of complex chromosomal rearrangements. The study included 16 unique complex rearrangements, of which 13 were resolved [21]. This work has since been extended into a national long‐read analysis pipeline, Nallo [22], developed by GMS‐RD in close collaboration with the National Genomics Infrastructure and Clinical Genomics platforms at SciLifeLab, providing a shared foundation for harmonised long‐read WGS analysis.

Building on these implementation efforts, GMS‐RD, in collaboration with the Wilhelm Foundation—a patient organization for undiagnosed diseases—initiated in 2023 the Undiagnosed Diseases Network Sweden (UDN Sweden) as a pathway for families remaining unsolved despite prior investigations. To date, >170 undiagnosed children have been included and analysed using short‐read trio WGS, long‐read WGS and short‐read RNA‐seq. Going forward, UDN Sweden will serve as an escalation pathway within GMS for the most challenging cases and enable systematic adoption of emerging technologies as well as a foundation for international collaboration.

**Fig. 3:**
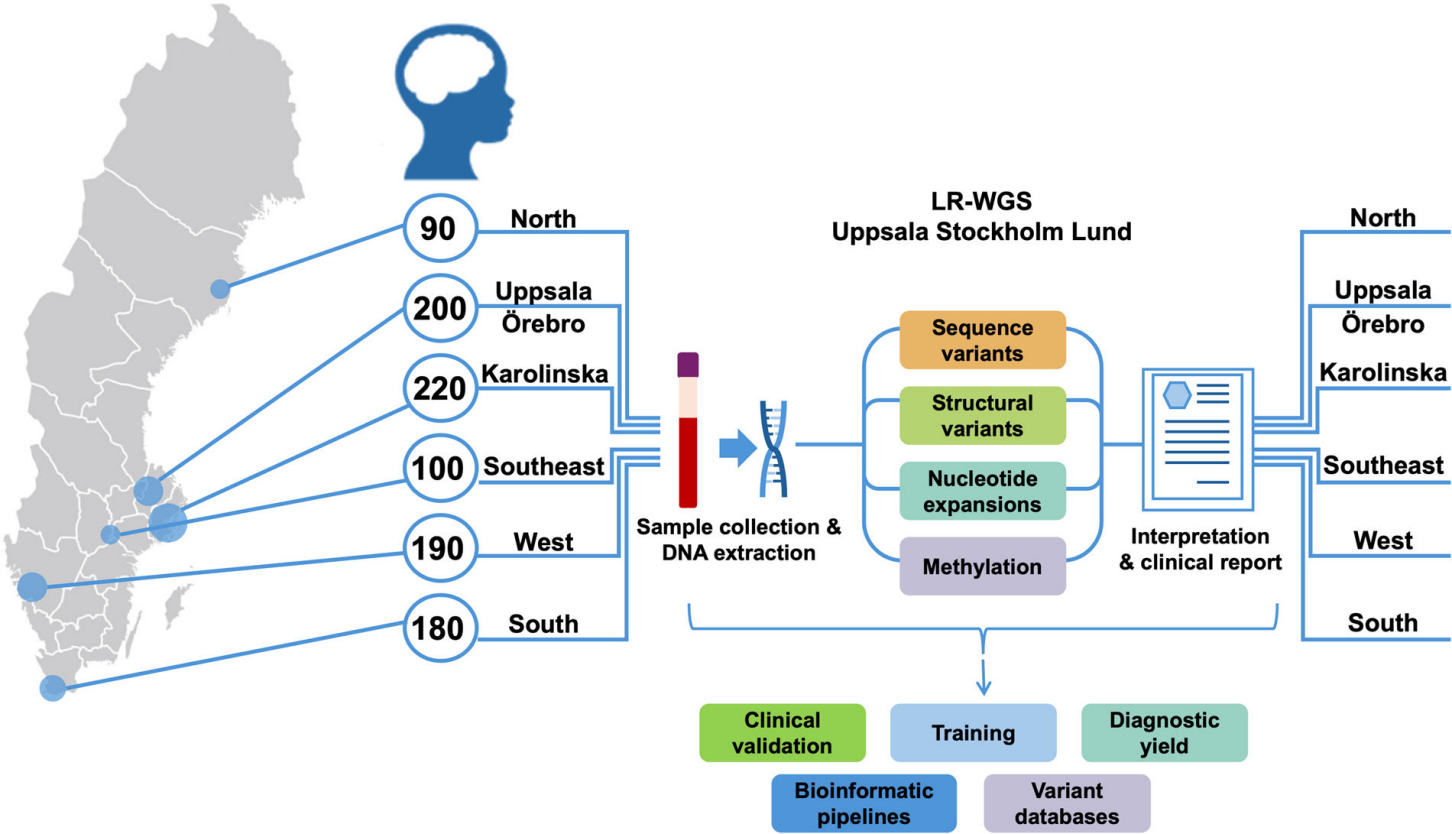
Ongoing national long‐read whole‐genome sequencing project (LRS1000+) in rare neurological diseases. All GMCs contribute patients according to population size, whereas long‐read sequencing is currently performed at three GMCs. LR‐WGS, long‐read whole‐genome sequencing.

The second GMS‐RD strategic project was launched in 2025 and aims to accelerate national transition to long‐read WGS by enrolling >1000 newly referred children and adults with rare neurological disorders (Fig. 3). In Phase 1, long‐read WGS is performed alongside short‐read WGS to build national capacity—that is, establish reference datasets, harmonize analysis and interpretation, develop QC and reporting standards and train teams across sites. In Phase 2, long‐read WGS will be implemented as a first‐line test for select patient groups if performance thresholds are met. The project is coordinated together with Genome of Sweden, a population‐based initiative aligned with the Genome of Europe (>1000 Swedish individuals), with data from both projects processed using the Nallo pipeline [22] and supported by shared QC and harmonized analytical standards. This marks a shift from pilots to coordinated implementation projects, where shared infrastructure and national collaboration enable rapid adoption of new technology.

### Haematological malignancies

Haematological malignancies comprise a diverse group of blood cancers characterized by marked clinical and biological heterogeneity and highly variable clinical outcomes [23, 24, 25]. These diseases are driven by a wide spectrum of genomic alterations, including SNV/indels, CNVs and complex SVs. Accurate and timely genomic profiling is essential for precise diagnosis, risk stratification, prognostication, selection of targeted therapies and disease monitoring, as reflected in contemporary disease classification and risk stratification systems [24, 26, 27, 28].

**Fig. 4:**
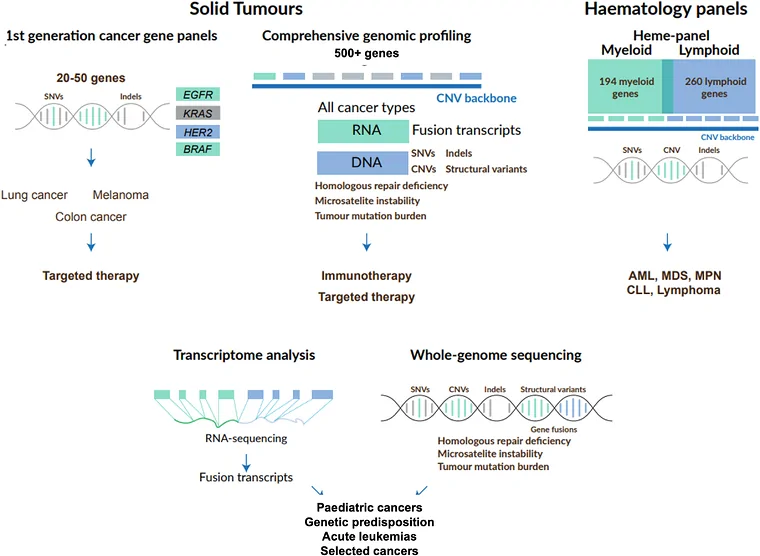
Genomic technologies used for cancer diagnostics in Sweden. National capture‐based gene panels are applied for solid tumours (upper left panel) and haematological malignancies (upper right panel). Whole‐transcriptome sequencing (WTS) and whole‐genome sequencing (WGS) (lower pan) are performed for childhood cancer, acute leukaemias and selected solid tumour indications. ALL, acute lymphoblastic leukaemia; AML, acute myeloid leukaemia; CLL, chronic lymphocytic leukaemia; CNVs, copy‐number variants; MPN, myeloproliferative neoplasias; SNVs, single nucleotide variants; SVs, structural variants.

The GMS‐Haematology Working Group focuses on harmonising and implementing standardized precision diagnostic approaches for haematologic malignancies across Sweden [7]. Important milestones include the design of a national capture‐based myeloid gene panel, including 191 genes recurrently found mutated in myeloid malignancies (Fig. 4) [29]. A similar lymphoid gene panel including 260 genes has also been developed [30]. These panels can be used separately or pooled in a single comprehensive assay (GMS‐HEME) and include a copy‐number backbone enabling detection of the most diagnostically important CNVs in myeloid and lymphoid malignancies.

To date, >22,000 samples have been analysed using the GMS myeloid gene panel. During development, the GMS panels were benchmarked against both commercial and non‐commercial gene panels with similar clinical applications. A major advantage of the captured‐based design is the ability to continuously adapt and update gene content in response to emerging scientific and clinical evidence, while maintaining full control over validation and quality assurance procedures.

In addition to targeted approaches, we have evaluated the performance of whole‐genome and transcriptome sequencing (WGTS) in haematological malignancies (Fig. 4). As a key strategic project, we have performed a national prospective study in adults and children with acute leukaemia (acute myeloid leukaemia and acute lymphoblastic leukaemia), to date including >700 cases from all 7 GMCs, where WGTS was conducted alongside standard‐of‐care (SoC) diagnostics [31, 32]. Based on these results, WGTS is now clinically offered to all children diagnosed with haematological malignancies with a required turnaround time (TAT) <2 weeks. Building on this work, a prospective real‐time validation study is currently underway in 250 adult cases with acute leukaemia, comparing SoC diagnostic approaches with WGTS. The study also assesses feasibility, TAT and health‐economic implications of implementing WGTS in routine practice. Collectively, these efforts will provide important evidence to guide the transition toward comprehensive genomic diagnostics of haematological malignancies.

Another important development has been the early recognition of the clinical significance of germline predisposition in haematological malignancies [33]. As WGS includes analysis of a matched normal sample, it enables identification of germline variants and accurate interpretation of tumour‐specific alterations. Germline predisposition is increasingly recognized, particularly in myeloid neoplasms, and current international guidelines recommend germline evaluation in selected patients. Accordingly, germline assessment has been incorporated into national diagnostic workflows, with clinical recommendations developed based on Nordic guidelines [33, 34, 35].

Overall, GMS‐Haematology has evaluated and implemented a range of genomic tests that enable comprehensive characterisation of haematological malignancies tailored to specific clinical indications. Ongoing and future efforts include harmonisation of bioinformatics pipelines, implementation of ultrasensitive sequencing‐based measurable residual disease detection, rapid identification of somatic variants using long‐read sequencing and gene expression‐based classification.

### Childhood cancer

The most common childhood cancers are leukaemias, followed by CNS tumours, whereas approximately one‐third represents non‐CNS solid tumours—a highly heterogeneous group of malignancies with a plethora of tumour subtypes. Accurate diagnosis based on morphology and immunohistochemistry can be challenging as many tumour types share an overlapping ‘small round blue cell’ morphology. The detection of somatic genetic changes has therefore been a tool in the diagnostic process for children with cancer for decades. However, with the advent of next‐generation sequencing (NGS), it became possible to comprehensively identify diagnostic and prognostic markers in paediatric tumours. In many paediatric cancer centres WGS has now become central to paediatric precision oncology, detecting the combined spectrum of SNVs, CNVs, SVs, tumour mutational burden (TMB) and mutational signatures beyond the scope of targeted panels (Fig. 4). Because many paediatric entities are fusion‑driven or show transcriptomic hallmarks, WTS complements WGS by detecting expressed fusions and splicing events.

For several types of paediatric cancer, WGTS has now been shown to largely reproduce results from SoC molecular tests while contributing additional disease‑relevant findings that influence management [36, 37, 38, 39, 40]. Sweden was among the first countries to establish a nationwide implementation program that offered WGTS to all children with a malignancy [41]. In this effort, GMS engaged all six paediatric oncology centres in a workflow integrating molecular pathology, clinical genetics and paediatric oncology. Initially launched as a national research program in 2021, WGTS transitioned to SoC in May 2024. A national harmonization group cross‑validates pipelines and aligns clinical reporting across laboratories to ensure consistent interpretation. During implementation (2021–2024), reporting time averaged 3 weeks, with ongoing efforts to reduce this to ≤2 weeks. To facilitate secondary research use of results, WGTS data collection and stewardship are coordinated through the Swedish Childhood Tumour Biobank (Barntumörbanken, BTB; https://www.barntumorbanken.se/).

Evidence for clinical utility in Sweden is so far based largely on retrospective data collection. In an initial nationwide cohort of 117 consecutively enrolled children with solid tumours, WGS (with RNA‑seq where indicated) identified clinically relevant alterations in 95% of tumours, adding diagnostic and/or prognostic information in about half of the cases [41]. In one‐third of the tumours, a potential treatment target was identified. However, the proportion of patients for whom the treatment was directly affected by the findings, for example, by medication with a precision drug, was less than 10%, similar to reports from other countries [37].

An essential component of the program has been the parallel analysis of germline and tumour DNA. Evaluation of germline data from 309 children with CNS and extracranial solid tumours identified pathogenic or likely pathogenic variants in childhood cancer predisposition genes in 11%; these findings led to tailored surveillance in 86% and treatment recommendations in 31% of patients [42]. Together with somatic tumour profiling, these results highlight the significant clinical value of comprehensive genome sequencing in paediatric oncology.

GMS‐Childhood Cancer is now developing a national data architecture within the NGP to integrate large‐scale data modalities. This integrated architecture, currently named BrainChild, aims to combine molecular, imaging and clinical data to support future research, including machine‐learning and artificial intelligence (AI)‐based studies.

### Solid tumours

The first drug targeting the HER2 protein (encoded by the *ERBB2* gene) in solid tumours was introduced at the turn of the century [43]. However, the development of anti‐EGFR therapies in colorectal and non‐small cell lung cancers made sequence‐based assays essential for therapeutic decision‐making [44, 45, 46]. With targeted therapies subsequently introduced at an increasing pace, the utility of limited targeted assays declined, and comprehensive genomic profiling (CGP) is more widely used to molecularly characterize major cancer forms for diagnostic, prognostic and treatment‐predictive purposes [47, 48, 49].

When the national molecular pathology collaboration Swedish Molecular Pathology transitioned to the GMS Solid Tumour Working Group (GMS‐ST) in 2017, NGS analyses using smaller amplicon‐based gene panels were already well established. GMS‐ST, therefore, designed a capture‐based panel allowing detection of complex biomarkers, such as microsatellite instability, TMB and homologous recombination deficiency (Fig. 4), while adding relevant genes for clinical purposes, trial inclusion and research use. The developmental work and strategic decisions on chemistry and design elements were aligned with work in GMS‐Haematology and solutions benchmarked against both commercial and non‐commercial gene panel alternatives. To allow for an effective detection of all treatment‐predictive fusion genes, an RNA design complemented the main design, forming the GMS560 DNA + RNA gene panel.

The selection of genes and biomarkers involved comprehensive consultation with national clinical and diagnostic reference groups. Candidate genes were also systematically scored based on consensus clinical and diagnostic literature, sequencing and clinical trial databases, established gene panel designs [49, 50], and in‐depth analyses of data from the Cancer Genome Atlas [51, 52]. In addition, genes relevant to pharmacogenomics and hereditary conditions were incorporated.

The GMS560 panel was developed through a joint national effort, analysing clinical samples with established biomarker status, aiming at a tool achieving conclusive results from the often scarce and formalin‐fixed paraffin‐embedded material available. In parallel, a bioinformatic pipeline, GMS Solid (https://github.com/genomic‐medicine‐sweden/GMS_Solid), was co‐developed to deliver the diverse set of variants and biomarkers crucial for interpretation. The panel was initially tested in national pilot studies supported by the government and has since been implemented at all GMCs, with >2000 analyses in 2025. It will also play a central role in FOCU.SE [47], an upcoming national drug repurposing trial that is part of the European PCM4EU and PRIME‐ROSE initiatives [53], and which will also serve to identify patients for other clinical trials.

Leveraging the flexibility inherent to a capture‐based design, GMS‐ST is currently updating the national panel design to incorporate novel biomarkers, while integrating design elements from the Genomic Medicine Centre Karolinska panel used in Stockholm. In the redesign, information from all WHO Blue Books on tumour classification was incorporated, together with mapping of key signal pathways [54, 55], and a more comprehensive pharmacogenomics component was added. This has resulted in a national panel (GMS Solid) that is up to date for treatment prediction and clinical trial inclusion, while also addressing novel diagnostic and prognostic questions.

In recent years, circulating tumour DNA (ctDNA) analysis has become increasingly important [56], prompting ongoing development of a ctDNA‐based version of the national gene panel for CGP to enable testing in cancers lacking representative tissue or cytological material. Future ctDNA applications include disease monitoring, detection of secondary resistance and measureable residual disease analyses.

Method development requiring validation in large cohorts with extensive clinical follow‐up—for example, gene expression analyses in select cancers—has not yet been prioritized but could be considered with additional funding. Instead, GMS‐ST has initiated pilots on global genomic analyses, including short‐read WGTS, initially implemented for sarcomas. Long‐read sequencing will initially be implemented for rapid methylation classification of CNS tumours [57] and later expanded to WGS across multiple tumour types, providing additional epigenetic and genetic information beyond short‐read sequencing.

### Microbiology

The inclusion of infectious diseases and clinical microbiology within GMS has resulted in a national framework for integrating advanced microbial genomics into clinical and public healthcare practice across Sweden. This enables consistent pathogen typing, antimicrobial resistance (AMR) surveillance and rapid responses to emerging infections [58, 59]. A suite of publicly available pipelines for the NGP supports national harmonization and reuse in microbiology (https://github.com/genomic‐medicine‐sweden).

A major pillar of GMS Microbiology has been the national rollout of long‑read 16S rRNA sequencing for bacterial identification and taxonomic classification directly from samples without prior culture. In a coordinated ring‑trial, samples (*n* = 20) were distributed to 20 laboratories (17/21 Swedish healthcare regions, the Public Health Agency of Sweden [PHAS], SciLifeLab and the Swedish Defence Research Agency), performing nanopore‐based, long‐read sequencing. All laboratories established the workflow and shared sequencing data within 2 months, which were jointly analysed using the commercial 1928 Diagnostics and the GMS TRANA 16S pipeline (https://github.com/genomic‐medicine‐sweden). This nationwide evaluation [60] paved the way for a broad harmonization and implementation, facilitating precision diagnostics of bacterial infections across most Swedish healthcare regions.

In parallel, GMS Microbiology has advanced a national strategy for WGS‐based surveillance, exemplified by a comprehensive methicillin‐resistant *Staphylococcus aureus* (MRSA) study [61]. Nine laboratories sequenced an MRSA panel from the PHAS national collection, consisting of 20 isolates from different strain transmissions, using local short‐read sequencing workflows. The raw data were analysed through three pipelines—JASEN, 1928 Diagnostics and CLC Genomics Workbench, Qiagen. Despite variation in sequencing depth and platforms, all isolates showed concordant clustering when analysed with the same pipeline. Hence, harmonized bioinformatic analysis of WGS data shared through the NGP can facilitate coordinated outbreak detection and AMR surveillance across health regions. An extended strategic project seeks to evaluate the benefits of sharing geographically linked sequencing data in real‐time using the GMS alert tool MIMOSA [62], allowing detection and alertness of cross‐regional transmissions and outbreaks. In this project, long‑read WGS data of all MRSA and extended‐spectrum beta‐lactamase‑producing *Klebsiella pneumoniae* isolates will be collected and shared over a 3‑month period across all 21 regions.

In addition, GMS has developed a broad metagenomics program to evaluate and align shotgun sequencing approaches for complex clinical samples, allowing detection of microorganisms (bacteria, fungi and viruses) directly from clinical samples. Eight laboratories have previously compared protocols for DNA/RNA extraction, human DNA depletion and short‑ and long‑read sequencing, with data analysis ongoing. During 2025, this work expanded into four strategic projects focusing on long‑read metagenomics across diverse disease areas, including sepsis, tuberculosis, orthopaedic infections and screening for faecal carriage of resistant microorganisms. These projects also assess TAT, diagnostic yield and the added value of long‑read sequencing for identifying species and AMR determinants.

Key priorities going forward include securing real‑time data sharing and connecting all remaining healthcare regions to establish fully national WGS and metagenomics capability. This broad national infrastructure for infectious disease genomics is also crucial for future international pandemic preparedness strategies.

### Pharmacogenomics

Pharmacogenomics aims to improve patient safety and therapeutic effectiveness by tailoring common medications to an individual's genes. These genes, referred to as pharmacogenes, influence drug metabolism, efficacy or susceptibility to serious adverse drug reactions. Analysis of 14 pharmacogenes in UK Biobank participants showed that 99.5% carried genetic variants potentially affecting response to at least one medication [63]. A European randomised trial of pre‑emptive testing for 12 pharmacogenes reported a 30% reduction in clinically relevant adverse drug reactions [64]. These results support broad implementation of pharmacogenomics in healthcare.

To support clinical translation, several international organizations provide standardized dosing guidelines [65, 66] that are available through the Clinical Pharmacogenomic Resource Database [67]. Many guidelines rely on the star‑allele system, which necessitates bioinformatic tools capable of interpreting diverse variant types. Such tools have been developed for pharmacogenetic star‐allele calling, most notably the bioinformatics pipeline and reporting tool Pharmacogenomics Clinical Annotation Tool (PharmCAT) [68].

Despite existing guidelines, pharmacogenetic testing in Sweden has up to now been limited. Clinical testing has focused on targeted detection of SNVs in well‑characterized pharmacogenes such as *CYP2C9*, *CYP2C19*, *TPMT* and *DPYD* [69]. These SNVs are used in routine PCR‑based assays testing and are sufficient for many established dosing guidelines.

However, increasing evidence highlights the importance of other variant types, including STRs, variable number tandem repeats (VNTRs), CNVs and complex SVs in pharmacogenomics. For example, the *UGT1A1* promoter TA‑repeat polymorphism is a determinant of irinotecan toxicity [70] that has been integrated into FDA‑endorsed dosing recommendations. Similarly, CNVs in the *CYP2D6* gene are essential for determining metabolizer status for many medications such as psychotropics and opioids [71]. The complex *CYP2D6* region includes deletions, duplications and hybrid structures that are difficult to detect using targeted genotyping or conventional short‑read sequencing [72].

Due to the complexity of many pharmacogenes, HTS offers advantages for pharmacogenomic testing [73]. Six pharmacogenes were included in the initial GMS cancer gene panels, and CPIC treatment recommendations were translated into Swedish. Recently, two GMCs developed an improved multigene NGS panel containing a curated set of over 40 clinically actionable pharmacogenes [74]. The capture‐based panel combined with short‑read sequencing is designed to detect SNVs, indels, SVs, STRs, VNTRs, CNVs and hybrid genes. The workflow includes in‑house bioinformatic analysis coupled with PharmCAT to generate standardized, guideline‑compatible dosing recommendations.

In 2025, GMS received funding from the government for national validation and implementation of the GMS Pharmacogenomics gene panel as well as for harmonized reporting and training of clinicians, geneticists, bioinformaticians and laboratory staff. Future work will focus on integrating pharmacogenetic data into electronic health records and generating evidence on the cost‐effectiveness of pharmacogenomic testing.

### Complex diseases

Complex diseases—such as cardiometabolic, respiratory, psychiatric, autoimmune conditions and cancer—are characterized by pronounced biological heterogeneity and multifactorial aetiology, involving interactions between polygenic risk, environmental exposures and lifestyle factors [75, 76]. These diseases typically lack a single ‘driver’ variant, limiting the explanatory power of genetics. Consequently, developing accurate disease prediction models based on genetic information has been challenging, and clinical use of genetics for diagnosis remains limited. One promising approach is polygenic risk scores (PRSs), which estimate cumulative genetic risk by aggregating the effects of thousands of variants. SNP genotyping provides a highly accurate and cost‐effective method to calculate PRS, and these scores can also be derived from WGS data [77].

Although PRSs have potential for risk prediction across many diseases, some areas are more immediately amenable to clinical implementation. For example, in breast cancer, up to 80%–90% of women with suspected familial forms never receive a molecular diagnosis (i.e., no high‐penetrance variant is detected). Many of these women exhibit elevated PRS, suggesting that familial aggregation may reflect polygenic genetics rather than single high‐penetrance variants [78]. This highlights the potential of PRS in clinical practice where they have been shown to improve risk stratification and thereby optimizing individualised management [79]. For breast, ovarian and prostate cancers, PRSs are already integrated into tools such as CanRisk (https://www.canrisk.org/), and discussions on implementing PRS for breast cancer are ongoing in Sweden. Beyond familial breast cancer, PRS may also support risk‐stratified population screening in the future [80]. Health‐economic evaluations suggest such approaches may provide substantial system‐level benefits if implemented in routine care [81].

Similar strategies apply to other diseases with both familial and polygenic components, such as cardiomyopathy and familial hypercholesterolaemia (FH). Hypertrophic cardiomyopathy (HCM), the most common inherited heart disease, involves abnormal thickening of the myocardium, leading to arrhythmias, heart failure and sudden cardiac death. Pathogenic variants in sarcomeric genes explain a proportion of HCM cases and inform diagnosis and clinical management. Yet at least 50% of HCM patients currently lack a genetic diagnosis. Recent studies indicate that PRS can help stratify such individuals at elevated risk [82]. Similarly, in suspected FH, only a subset of patients carries known high‐penetrance variants. More than half of FH variant carriers remain disease‐free into middle age, partly due to incomplete penetrance influenced by polygenic background [83]. Importantly, the most clinically consequential outcome of hypercholesterolaemia is coronary artery disease (CAD), the underlying pathology leading to myocardial infarction. PRSs for CAD, where individuals in the top 5% have approximately fivefold higher risk [84], provide a complementary framework for improved risk‐stratification of individuals with elevated LDL and support more precise prevention strategies.

Alongside genomic variation, the complex disease field is increasingly enriched by results from other large‐scale omics technologies, particularly proteomics and metabolomics. These molecular layers can often be measured from minimally invasive samples and increasingly at scale in population cohorts, providing a dynamic snapshot of physiological and metabolic states. Recent studies have demonstrated the potential of circulating protein signatures [85, 86] and metabolomic profiles [87, 88] for early disease detection across multiple conditions. Integrating these modalities with genetics also enables identification of causal relationships [89].

Although a small number of biomarkers are beginning to enter clinical use [90], most findings remain at the discovery stage. Future progress will depend on substantially larger cohorts and longitudinal follow‐up to establish robust associations, causal mechanisms and predictive value. To support this, the Precision Omics Initiative Sweden—launched by GMS and SciLifeLab—integrates multi‐omics data with population resources and long‐term health outcomes to explore molecular signatures and their clinical potential [91].

## Bioinformatics as a core pillar of precision diagnostics

Genomics‐based precision diagnostics utilizing NGS enable detailed characterization of genomic aberrations but generate vast amounts of sequence data that must be processed through multiple steps before clinical interpretation can yield actionable insights. The main steps of the process for germline (RD) and somatic (cancer) analyses include (1) read alignment to a reference genome, (2) variant calling to identify different categories of genomic aberrations, (3) annotation with information on genomic features, functional consequences, population frequencies and pathogenicity predictions, and (4) variant prioritization or filtering step to generate a short list for clinical interpretation.

To support the diagnostic working groups, we have developed bioinformatic analysis workflows for all major disease areas, often building on the standards developed by the global bioinformatic community. Examples include the modular workflows written in Nextflow and developed within the nf‐core community [92] and the hydra‐genetics workflows written in Snakemake (https://github.com/hydra‐genetics/). Key features of the GMS‐developed workflows include comprehensive functionality for processing data to a short list of variants for interpretation; high quality of programmatic coding, including unit and integration tests; transparent modular code; documentation and a structured development strategy linking user requirements to feature development and performance verifications and validations. All workflows are publicly available via the code‐sharing repository GitHub (https://github.com/genomic‐medicine‐sweden). The tools adhere to open‐source principles, enabling reproducibility and community‐driven enhancement. Workflow development also follows established principles for clinical bioinformatics [93].

To support clinical interpretation, dedicated data interpretation solutions are being developed to match the clinical needs and enable efficient interpretation that also captures and structures the interpretation results. For RD, including germline cancer predisposition, Scout [5] serves as a central portal for interpretation, providing access to prioritised variants, quality reports and links to internal and external knowledge bases. Scout also links to additional tools supporting clinical interpretation, including, for example, GENS (https://github.com/SMD‐Bioinformatics‐Lund/gens) for interactive evaluation of CNVs.

Together, these bioinformatic workflows and interpretation tools—developed under the GMS umbrella—strengthen collaboration between clinical and bioinformatic experts, are increasingly used across GMCs and support national harmonization of genomic‐based precision diagnostics.

## The National Genomics Platform

Currently, computational infrastructures for processing genomic data remain distributed across the different GMCs. Going forward, GMS is complementing this with a large‐scale national informatics infrastructure, the NGP, for storage, analysis and sharing of genomic data within Swedish healthcare. The infrastructure supports improved interoperability, scalability and data exchange in both diagnostic and research settings. Inter‐regional agreements executed between Region Västra Götaland (which operates the NGP) and the partnering regions and universities formalise governance and technical connectivity for secure data transfer through national healthcare networks. These agreements reflect the collaborative governance model underpinning the platform's integration into Swedish healthcare.

**Fig. 5:**
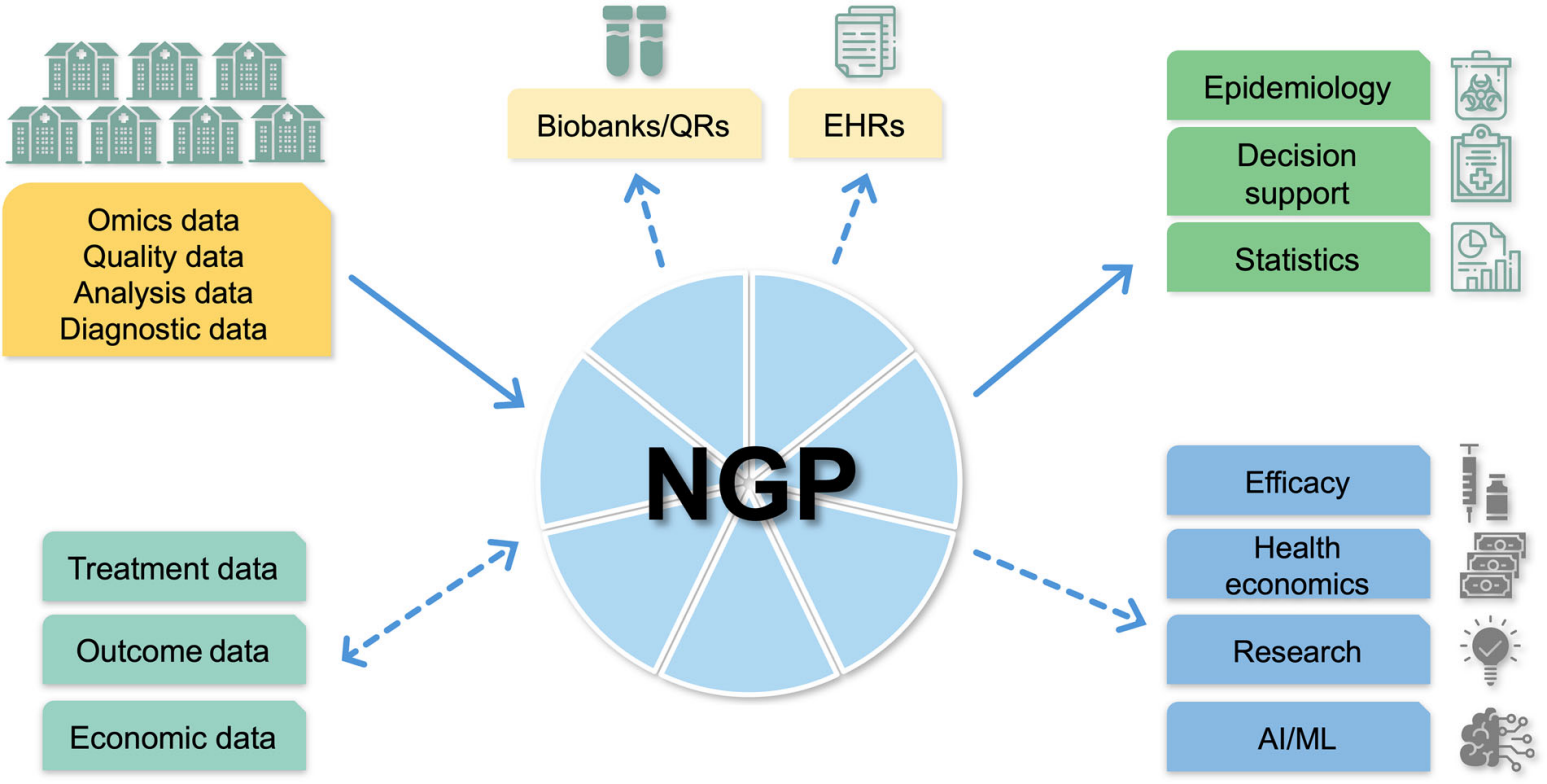
The National Genomics Platform (NGP) serves as the central infrastructure of Genomic Medicine Sweden (GMS) for genomic data storage, computing and secure data sharing. Each university healthcare region operates within its own tenant in the NGP, ensuring regional governance and control of data. Genomic data and associated metadata are uploaded from participating sites to the NGP according to national standards. The platform supports multiple uses of the data, including quality assurance, variant data sharing and health‐economic analyses. Ongoing integrations include connections to national cancer quality registries and relevant governmental agencies. AI, artificial intelligence.

The NGP serves several critical functions covering computation, storage, analysis and secondary use of data. It also includes specialized portals for feasibility studies aimed at attracting new clinical studies to Sweden and a variant database portal that provides both a knowledge base and quality assurance for diagnostics performed across Sweden. Data and associated metadata are stored and structured according to national standards. Architecturally, the NGP implements isolated tenants for each GMC, ensuring that each region retains full control over its own data but at the same time allowing selected data to be shared within the NGP (Fig. 5). This separation extends across storage, indexing and computation layers, enabling fine‐grained governance over shared metadata and interpretations. Data analysis using the jointly developed bioinformatic workflows operates across the platform to detect clinically relevant genomic variants. NGP also enables secure data sharing within ethical and regulatory frameworks, currently limited to approved research projects, while laying the foundation for future clinical data interoperability as legislation evolves.

## Legal and regulatory aspects

The implementation of genomic‐based precision medicine at national scale requires a robust and adaptive legal framework. Within GMS, legal expertise has, therefore, been engaged as a cross‐cutting function aligned with the initiative's core activities. A central component of this work has been continuous legal analysis of ongoing and planned GMS activities. New diagnostic workflows, data infrastructures, research collaborations and national pilots have been assessed against applicable national and EU legislation. This has ensured that implementation has occurred within existing legal frameworks.

GMS comprises 14 separate legal entities—seven healthcare regions and seven universities—each with independent legal responsibility. National coordination, therefore, requires a comprehensive contractual structure. A substantial body of inter‐regional agreements has been developed to regulate responsibilities for data processing, infrastructure access and governance within the NGP. Additional agreements cover specific projects. Collaboration with external stakeholders also requires structured agreements to ensure compliance with public law.

Beyond compliance and contractual governance, GMS has contributed to shaping the broader legal landscape. Experience implementing precision medicine within a regionally organized healthcare system has highlighted limitations in current legislation, particularly for cross‐regional clinical data sharing. To address these challenges, GMS submitted a petition to the Swedish government requesting updated legislation to enable secure and proportionate data sharing in precision medicine. Notably, a legal expert affiliated with GMS was also appointed to lead a governmental inquiry on strengthening legal conditions for expanded secondary use of health data. This work contributes to aligning national legislation with emerging European frameworks, including the European Health Data Space.

The regulatory landscape has also created challenges, particularly due to the impact of the In Vitro Diagnostic Regulation (2017/746). In‐house developed devices under Article 5 [5] may not be transferred to another legal entity, which limits the ability of multiple regions to jointly own or distribute products developed together. Collaboration during the development phase is possible; however, each legal entity must subsequently take independent responsibility for the manufacture, documentation, validation and regulatory compliance of the in‐house device.

Considering these challenges, continued attention and further development will be required, particularly regarding the legal infrastructure supporting precision medicine. The integration of legal expertise within GMS ensures responsible action within existing regulatory frameworks while laying the foundation for the future development of precision medicine and a sustainable healthcare system.

## Health economics

The Swedish healthcare system is publicly funded, with limited patient co‐payment for care and pharmaceuticals [94]. Although implementation of genomic medicine raises important health‐economic considerations, these technologies often involve high upfront costs not routinely encountered by healthcare providers but may generate long‐term health gains and future cost savings. Rigorous economic evaluations are therefore essential to determine whether genomic interventions produce net health gains within a resource‐constrained system. Thus far, cost‐effectiveness has rarely been systematically considered in decisions on implementing genomics‐based diagnostics in Sweden [95].

A key task for the GMS Health Economics Working Group is therefore to assess whether the implementation of genomics in routine care is likely to be cost‐effective. However, cost‐effectiveness alone is often insufficient to ensure equitable and sustainable implementation. Assessing the impact on healthcare budgets is equally important. Interventions that appear cost‐effective over a lifetime may still place substantial pressure on annual healthcare budgets. Careful assessment of affordability and budget impact is therefore important to mitigate the risk of crowding out other high‐value care.

The working group will evaluate the cost‐effectiveness of pharmacogenomic testing in solid tumours and WGTS in acute leukaemias compared with current SoC. Although these studies focus on Sweden, the methodological challenges of evaluating genomic medicine are universal. Uncertainty regarding long‐term outcomes, rapidly evolving technologies, small patient populations and the integration of companion diagnostics can challenge traditional economic evaluation frameworks [96]. This creates strong opportunities for international collaboration, such as the Beyond 1 Million Genomes Plus project (https://b1mgplus.onemilliongenomes.eu/), where we are actively involved, to develop methodological standards, share evidence and harmonize best practices for the economic evaluation of genomics‐driven healthcare.

## Strategic partnerships and precompetitive collaboration in precision medicine

GMS has established collaborations with several national genomic/precision medicine initiatives, including those in Denmark, England, France, Germany and the Netherlands. Such international collaborations and strategic partnerships are essential for advancing precision medicine, particularly in a country the size of Sweden, by enabling knowledge exchange, harmonization and the shared development of best practices.

Moreover, precompetitive collaboration between healthcare, academia and industry is crucial for the development and implementation of precision medicine at a national level [97]. GMS partners and national life science trade associations have agreed on a collaboration framework to address legal issues, strengthen cooperation and accelerate the healthcare system's transition toward patient‑centred precision diagnostics and medicine.

Strategic industry collaboration is an integrated part of GMS operations and focuses on value creation aligned with the overall goals. For example, in a joint effort with the National Lung Cancer Registry and Amgen Sweden AB, a population‐based study of 6000 lung cancer patients was conducted, showing that patients with the KRAS G12C mutation have a high risk of brain metastases [98]. This work improved understanding of disease progression and opportunities for individualized treatment.

Several large‐scale strategic projects focusing on short‐ and long‐read WGS/WTS have been conducted in close collaboration with industry. These partnerships provide access to the latest technological developments, as many collaborators are global companies at the forefront of innovation. During refinement of the GMS560 panel, companies were invited to confidentially provide input on upcoming treatments requiring genomic diagnostics and anticipated needs for planned clinical trials.

Clinical studies and trials are important steps in implementation of new diagnostic methods and treatments in precision medicine. To facilitate this, a feasibility portal is being developed within the NGP, enabling companies and researchers to determine the number of potentially eligible patients based on predefined criteria—such as specific gene mutations—for planned clinical trials.

Collaboration is built on trust and mutual benefits, and developing strong relationships requires time and commitment. Although challenges such as lengthy negotiations and potential conflicts of interest may arise, stakeholders can still work toward shared goals. Clear processes and dedicated collaboration facilitators are important success factors. Precompetitive collaboration between healthcare, academia and industry will remain essential for accelerating innovation and implementing precision medicine nationwide.

## Competence development across healthcare and related professions

In Sweden, as in many other countries, the shortage of healthcare professionals trained in genomic medicine (e.g., clinical geneticists and genetic counsellors) remains a major barrier to the integration of genomic medicine into routine care.

In 2022, GMS conducted a survey among non‐genetics physicians in Sweden to assess their knowledge and skills in genomic medicine, and preferences for competence development were assessed [99, 100]. Results indicated that physicians were already engaged with genomic medicine to a large extent but reported limited confidence in their knowledge and skills. The survey also showed strong interest in further training, highlighting the need for continuing professional development. A large majority (74%) reported that they had received too little education in genomic medicine during their university training. However, most responders (82%) considered genomic medicine to be more suitable for continuous professional education than for undergraduate medical training. Nevertheless, we anticipate that genomic medicine will also need to be more effectively integrated into medical education curricula.

To support competence development in genomic medicine, GMS runs regular webinars for the healthcare profession aimed at building knowledge of genomic methods, precision diagnostics and emerging applications. The series also includes patient stories, providing deeper insight not only into the technological advances of precision medicine but also into the areas where patients themselves perceive the greatest benefit. More than 600 participants attend monthly seminars each year, with several hundred additional viewers accessing each seminar via the GMS YouTube channel.

GMS also partners with universities, industry and patient organisations to develop educational materials in precision medicine, including a massive open online course ‘Introduction to precision medicine’ and an e‐learning module ‘Precision medicine in breast cancer’. Ensuring equitable global access to genomic and precision medicine requires strengthened education and training of the healthcare workforce. To contribute to this effort, GMS participates in the Global Genomics Network for Education and Training (https://ggnet‐genomics.com/).

Future efforts include improving general literacy in genetics and genomic testing, as well as awareness of associated ethical considerations. In addition, genomic and precision medicine will need to be better integrated into specialist training across multiple medical specialties to ensure appropriate use and interpretation of genomic diagnostics. We also recognise the need for continued efforts to strengthen competence across all healthcare regions, including those outside the university hospital settings. Here, the regional GMCs play an important role in supporting broader implementation. Together with national stakeholders, GMS will continue to develop and adapt educational and training resources for the Swedish healthcare workforce.

Finally, a substantial shortage of expertise in IT, bioinformatics and clinical variant interpretation remains. Building, expanding and securing this competence through sustained education, training and recruitment initiatives will be essential for the long‐term implementation and scalability of precision medicine.

## Empowering the development of precision medicine with the patient voice

Engagement with patient representatives and patient organizations has been integral to GMS since its inception. This includes representatives from the two nationwide umbrella patient organizations for cancer and RD—Network Against Cancer and Rare Diseases Sweden—serving on the GMS National Steering Board and contributing to the operational work through the GMS Patient Cooperation Council. This collaboration highlights the importance of patient involvement in advancing precision medicine. Activities include integrating the patient perspective into seminars and dialogues with healthcare professionals and policymakers; developing accessible information materials on genomic medicine for patients, next‐of‐kin and healthcare professionals; and establishing eConsents.

GMS aims to further strengthen cooperation and co‐development with patients and patient representatives through dialogue forums advising leadership on issues such as consent, data sharing and communication. As genomic and precision medicine increasingly become part of routine care, GMS will also engage with the public to better understand attitudes and needs.

## Concluding remarks and future directions

Over the last 10–15 years, sequencing‐based technologies have evolved from primarily research‐driven tools into an essential component of clinical diagnostics, enabling more accurate diagnoses, refined risk stratification, and increasingly individualized care across RD, cancer, infectious diseases and pharmacogenomics. Sweden's experience through GMS demonstrates how a regionally organised healthcare system can implement genomics‐based precision medicine at scale by combining strong national coordination, regionally embedded GMCs, disease‐specific expert groups and shared technical infrastructure [13, 16]. Harmonised clinical workflows, community‐driven bioinformatics and cross‐sector partnerships have enabled early national roll‐out of high‐impact applications such as WGS in RD diagnostics, WGTS in paediatric cancer, large gene panels for haematological and solid tumours, and genomics‐enabled microbiological surveillance. Nevertheless, several challenges remain, including legal complexity related to data sharing, long‐term funding, competence provision and ensuring equitable implementation and access across the country.

Looking ahead, the next phase of genomic medicine in Sweden will be defined less by technological availability and more by sustainable, harmonised integration into routine care. Key priorities include ensuring equitable access across all healthcare regions, achieving TATs that meet clinical needs, and generating robust evidence of clinical utility and cost‐effectiveness to support long‐term adoption. In this context, the recently launched national roadmap for the implementation of precision medicine in healthcare is an important step forward [101]. In addition, the Swedish government is initiating a 5‐year national program aimed at supporting the equal implementation of precision diagnostics across the country. Furthermore, as longer‐term follow‐up data are available, we plan to systematically evaluate the impact of precision diagnostics approaches on clinical outcomes across patient groups in a comprehensive and unbiased manner.

GMS will play a key role in strengthening research and innovation in genomic‐based precision medicine by enabling access to harmonised data resources, shared analytical infrastructure and coordinated national and international collaboration. Continued national alignment of laboratory methods, bioinformatics pipelines and variant interpretation practices will be critical to ensure robust, scalable and clinically consistent implementation of precision diagnostics across Sweden. This is particularly important as diagnostics increasingly transition toward comprehensive approaches (e.g., WGTS and long‐read sequencing) and emerging analyte types (e.g., ctDNA, methylation and metagenomics) become integrated into routine clinical care. In particular, sustained development, validation and standardization of bioinformatics pipelines will be essential to support reproducibility, interoperability, regulatory compliance and equitable access to high‐quality genomic diagnostics nationwide. Our activities will be coordinated with the Precision Medicine Centres, recently established at university hospitals, and with a strategic role in the broader, technology‐agnostic implementation of precision medicine in healthcare.

A major enabling step will be the maturation of the NGP into a scalable environment for secure storage, analysis and secondary use of genomic data. As governance and legislation evolve, Sweden will be well‐positioned to move toward a cohesive national learning healthcare system, in which aggregated data support reanalysis, method development, AI‐enabled interpretation and rapid translation of new evidence into practice. In parallel, strengthening the genomic medicine workforce and improving genomic literacy among non‐genomics healthcare professionals will be critical for the broad integration of genomic medicine across the country.

Finally, sustained patient involvement will support trust and participation, whereas strategic partnerships with industry and SciLifeLab will accelerate innovation, clinical trials and the implementation of emerging technologies. Together, these efforts will position Sweden to broaden the clinical impact of precision medicine by shortening diagnostic journeys, enabling more precise treatment selection, strengthening outbreak preparedness, supporting risk‐stratified prevention and ultimately enabling early detection of disease while maintaining the principles of equity within a publicly funded healthcare system.

## Author contributions

**Anna Lindstrand**: Conceptualization; writing—original draft; writing—review and editing; visualization. **Maria Johansson Soller**: Conceptualization; writing—review and editing. **Åsa Johansson**: Conceptualization; writing—original draft; writing—review and editing. **Malin Melin**: Conceptualization; writing—review and editing. **Anna Wedell**: Conceptualization; writing—review and editing. **Mikaela Friedman**: Conceptualization; writing—original draft; writing—review and editing. **Richard Rosenquist**: Conceptualization; writing—original draft; writing—review and editing; visualization; funding acquisition. **Lucia Cavelier**: Conceptualization; writing—original draft; writing—review and editing; visualization. **Mia Wadelius**: Conceptualization; writing—original draft; writing—review and editing. **Panagiotis Baliakas**: Conceptualization; writing—original draft; writing—review and editing. **Christian G. Giske**: Conceptualization; writing—original draft; writing—review and editing. **Hans Ehrencrona**: Conceptualization; writing—original draft; writing—review and editing. **Lovisa Lovmar**: Conceptualization; writing—original draft; writing—review and editing. **Anders Edsjö**: Conceptualization; writing‐original draft; writing‐review and editing; visualization; funding acquisition. **David Gisselsson**: Conceptualization; writing—original draft; writing—review and editing. **Fulya Taylan**: Conceptualization; writing—review and editing. **Hannes Olauson**: Conceptualization; writing—original draft; writing—review and editing. **Henrik Green**: Conceptualization; writing—original draft; writing—review and editing. **Markus Heidenblad**: Conceptualization; writing—review and editing. **Paula Mölling**: Conceptualization; writing—original draft; writing—review and editing. **Anna Green**: Conceptualization; writing—review and editing; writing—original draft. **Erika Tång Hallbäck**: Conceptualization; writing—original draft; writing—review and editing. **Anders Jemt**: Conceptualization; writing—original draft; writing—review and editing. **Jens Enoksson**: Conceptualization; writing—review and editing. **Magnus Sabel**: Conceptualization; writing—review and editing. **Carl Mårten Lindqvist**: Conceptualization; writing—review and editing. **Kina Höglund**: Conceptualization; writing—review and editing. **Martin Bergö**: Conceptualization; writing—review and editing. **Craig E. Wheelock**: Conceptualization; writing—review and editing. **Ann Ekberg Jansson**: Writing—review and editing; conceptualization. **Ann Nordgren**: Conceptualization; writing—original draft; writing—review and editing. **Cecilia Fagerström**: Conceptualization; writing—review and editing. **Hartmut Vogt**: Conceptualization; writing—review and editing. **Stephanie Anja Juran**: Conceptualization; writing—review and editing. **Mats G. Karlsson**: Conceptualization; writing—review and editing. **Jan Holgersson**: Conceptualization; writing—review and editing. **Martin Hallbeck**: Conceptualization; writing—review and editing. **Frida Lundmark**: Conceptualization; writing—review and editing. **Colum Walsh**: Conceptualization; writing—review and editing. **Malin Sund**: Conceptualization; writing—review and editing. **Sofia Gruvberger‐Saal**: Conceptualization; writing—review and editing. **Marene Landström**: Conceptualization; writing—review and editing. **Eva Tiensuu Janson**: Conceptualization; writing—review and editing. **Richard Palmqvist**: Conceptualization; writing—review and editing. **Maria Johansson**: Conceptualization; writing—original draft; writing—review and editing. **Lars Palmqvist**: Conceptualization; writing—review and editing. **Päivi Östling**: Conceptualization; writing—review and editing. **Per Sikora**: Conceptualization; writing—review and editing; visualization. **Andreas Muranyi Scheutz**: Conceptualization; writing—review and editing. **Bianca Stenmark**: Conceptualization; writing—review and editing. **Katarina Nyström**: Conceptualization; writing—review and editing; writing—original draft. **Tobias Strid**: Conceptualization; writing—review and editing. **Malin Karnå**: Conceptualization; writing—review and editing. **Oskar Frisell**: Conceptualization; writing—original draft; writing—review and editing. **Mirja Carlsson Möller**: Conceptualization; writing—original draft; writing—review and editing; project administration. **Thoas Fioretos**: Conceptualization; writing—original draft; writing—review and editing. **Mats Ulfendahl**: Conceptualization; writing—review and editing. **Valtteri Wirta**: Conceptualization; writing—original draft; writing—review and editing. **Therese Fagerqvist**: Conceptualization; writing—original draft; writing—review and editing.

## Use of artificial intelligence (AI)

ChatGPT (OpenAI) was used to support language editing and stylistic improvements. All outputs were reviewed and verified by the authors.

## Conflict of interest statement

A.E. has received honouraria from Amgen, AstraZeneca, Bayer, Diaceutics, Pfizer and Roche. A.L. has received speakers’ honouraria from Pacific Biosciences and Illumina. A.N. is a member of the KI‐Biogen Joint Advisory Committee, SmartCella Scientific Advisory Board and board‐member of Sällsyntafonden, Ågrenska AB (svb) and Sävstaholm foundation. T.F.I. has received honouraria from Illumina, serves as a scientific advisor to Qlucore AB and Cantargia AB and is a board member of Lead Biologics Intl. R.R. has received honouraria from AbbVie, AstraZeneca, Illumina, Janssen, Eli Lilly and Roche. The other authors declare no conflicts of interest.

## Data Availability

Data sharing is not applicable to this article as no datasets were generated or analysed during the current study.
